# Intelligent digital tools for screening of brain connectivity and dementia risk estimation in people affected by mild cognitive impairment: the AI-Mind clinical study protocol

**DOI:** 10.3389/fnbot.2023.1289406

**Published:** 2024-01-05

**Authors:** Ira H. Haraldsen, Christoffer Hatlestad-Hall, Camillo Marra, Hanna Renvall, Fernando Maestú, Jorge Acosta-Hernández, Soraya Alfonsin, Vebjørn Andersson, Abhilash Anand, Victor Ayllón, Aleksandar Babic, Asma Belhadi, Cindy Birck, Ricardo Bruña, Naike Caraglia, Claudia Carrarini, Erik Christensen, Americo Cicchetti, Signe Daugbjerg, Rossella Di Bidino, Ana Diaz-Ponce, Ainar Drews, Guido Maria Giuffrè, Jean Georges, Pedro Gil-Gregorio, Dianne Gove, Tim M. Govers, Harry Hallock, Marja Hietanen, Lone Holmen, Jaakko Hotta, Samuel Kaski, Rabindra Khadka, Antti S. Kinnunen, Anne M. Koivisto, Shrikanth Kulashekhar, Denis Larsen, Mia Liljeström, Pedro G. Lind, Alberto Marcos Dolado, Serena Marshall, Susanne Merz, Francesca Miraglia, Juha Montonen, Ville Mäntynen, Anne Rita Øksengård, Javier Olazarán, Teemu Paajanen, José M. Peña, Luis Peña, Daniel lrabien Peniche, Ana S. Perez, Mohamed Radwan, Federico Ramírez-Toraño, Andrea Rodríguez-Pedrero, Timo Saarinen, Mario Salas-Carrillo, Riitta Salmelin, Sonia Sousa, Abdillah Suyuthi, Mathias Toft, Pablo Toharia, Thomas Tveitstøl, Mats Tveter, Ramesh Upreti, Robin J. Vermeulen, Fabrizio Vecchio, Anis Yazidi, Paolo Maria Rossini

**Affiliations:** ^1^Department of Neurology, Oslo University Hospital, Oslo, Norway; ^2^BrainSymph AS, Oslo, Norway; ^3^Memory Clinic, Fondazione Policlinico Universitario Agostino Gemelli IRCCS, Rome, Italy; ^4^Department of Neuroscience, Catholic University of the Sacred Heart, Rome, Italy; ^5^Department of Neuroscience and Biomedical Engineering, Aalto University, Helsinki, Finland; ^6^BioMag Laboratory, HUS Medical Imaging Centre, Helsinki University Hospital, Helsinki University and Aalto University School of Science, Helsinki, Finland; ^7^Centre for Cognitive and Computational Neuroscience, Universidad Complutense de Madrid, Madrid, Spain; ^8^Department of Experimental Psychology, Cognitive Psychology and Speech and Language Therapy, Universidad Complutense de Madrid, Pozuelo de Alarcón, Spain; ^9^Institute of Sanitary Investigation (IdISSC), San Carlos University Hospital, Madrid, Spain; ^10^Center for Computational Simulation, Universidad Politécnica de Madrid, Madrid, Spain; ^11^Performance and Assurance Solutions, Digital Solutions, DNV, Oslo, Norway; ^12^Lurtis Rules S.L., Madrid, Spain; ^13^Healthcare Programme, Group Research and Development, DNV, Oslo, Norway; ^14^Department of Computer Science, OsloMet—Oslo Metropolitan University, Oslo, Norway; ^15^NordSTAR—Nordic Center for Sustainable and Trustworthy AI Research, Oslo, Norway; ^16^Alzheimer Europe, Luxembourg, Luxembourg; ^17^Department of Radiology, Universidad Complutense de Madrid, Madrid, Spain; ^18^Department of Neuroscience and Neurorehabilitation, IRCCS San Raffaele, Rome, Italy; ^19^Pre Diagnostics AS, Oslo, Norway; ^20^The Graduate School of Health Economics and Management (ALTEMS), Catholic University of the Sacred Heart, Rome, Italy; ^21^IT Department, University of Oslo, Oslo, Norway; ^22^Department of Geriatric Medicine, Hospital Universitario Clínico San Carlos, Madrid, Spain; ^23^Department of Geriatrics, Fundación para la Investigación Biomédica del Hospital Clínico San Carlos, Madrid, Spain; ^24^Department of Medical Imaging, Radboud University Medical Center, Nijmegen, Netherlands; ^25^Division of Neuropsychology, HUS Neurocenter, Helsinki University Hospital and Helsinki University, Helsinki, Finland; ^26^Department of Neurology, Helsinki University Hospital and Clinical Neurosciences, Neurology, University of Helsinki, Helsinki, Finland; ^27^Department of Computer Science, Helsinki Institute of Information Technology, Aalto University, Helsinki, Finland; ^28^Department of Computer Science, University of Manchester, Manchester, United Kingdom; ^29^Department of Neurosciences, University of Helsinki, Helsinki, Finland; ^30^Neurocenter, Neurology, Kuopio University Hospital, Kuopio, Finland; ^31^Neurology Department, Hospital Universitario Clínico San Carlos, Madrid, Spain; ^32^The Norwegian Health Association, Oslo, Norway; ^33^Neurology Service, Hospital General Universitario Gregorio Marañón, Madrid, Spain; ^34^Finnish Institute of Occupational Health, Helsinki, Finland; ^35^School of Digital Technologies, Tallinn University, Tallinn, Estonia; ^36^Memory Unit, Department of Geriatrics, Hospital Clínico San Carlos, Madrid, Spain; ^37^Institute of Clinical Medicine, University of Oslo, Oslo, Norway; ^38^Department of Theoretical and Applied Sciences, eCampus University, Como, Italy

**Keywords:** mild cognitive impairment, dementia, machine learning, artificial intelligence, electroencephalography (EEG), magnetoencephalography (MEG), clinical study protocol, AI-Mind

## Abstract

More than 10 million Europeans show signs of mild cognitive impairment (MCI), a transitional stage between normal brain aging and dementia stage memory disorder. The path MCI takes can be divergent; while some maintain stability or even revert to cognitive norms, alarmingly, up to half of the cases progress to dementia within 5 years. Current diagnostic practice lacks the necessary screening tools to identify those at risk of progression. The European patient experience often involves a long journey from the initial signs of MCI to the eventual diagnosis of dementia. The trajectory is far from ideal. Here, we introduce the AI-Mind project, a pioneering initiative with an innovative approach to early risk assessment through the implementation of advanced artificial intelligence (AI) on multimodal data. The cutting-edge AI-based tools developed in the project aim not only to accelerate the diagnostic process but also to deliver highly accurate predictions regarding an individual's risk of developing dementia when prevention and intervention may still be possible. AI-Mind is a European Research and Innovation Action (RIA H2020-SC1-BHC-06-2020, No. 964220) financed between 2021 and 2026. First, the AI-Mind *Connector* identifies dysfunctional brain networks based on high-density magneto- and electroencephalography (M/EEG) recordings. Second, the AI-Mind *Predictor* predicts dementia risk using data from the *Connector*, enriched with computerized cognitive tests, genetic and protein biomarkers, as well as sociodemographic and clinical variables. AI-Mind is integrated within a network of major European initiatives, including The Virtual Brain, The Virtual Epileptic Patient, and EBRAINS AISBL service for sensitive data, HealthDataCloud, where big patient data are generated for advancing digital and virtual twin technology development. AI-Mind's innovation lies not only in its early prediction of dementia risk, but it also enables a virtual laboratory scenario for hypothesis-driven personalized intervention research. This article introduces the background of the AI-Mind project and its clinical study protocol, setting the stage for future scientific contributions.

## 1 Introduction

Dementia affects nearly 10 million new individuals every year, with Alzheimer's disease (AD) dementia accounting for about 60–80% of cases (Roberts et al., 2014). Crucially, AD and other forms of dementia types represent a growing global crisis, particularly in regions where life expectancy has risen notably over recent decades (GBD 2019 Dementia Forecasting Collaborators, 2022). The original description of AD is based on macroscopic brain atrophy, primarily behind the central sulcus and within the mesiotemporal regions. These changes are typically associated with memory deficits. Additionally, pathophysiological markers include extracellular amyloid plaques, intracellular neurofibrillary tangles, and synaptic loss. Importantly, the time from memory disorder diagnosis of any type to the death of a patient has increased significantly due to more timely diagnostic assessments and better medical and social care, especially in developed countries (Livingston et al., 2020). Consequently, dementia now imposes a substantial socioeconomic burden on health systems, families, and caregivers (Jetsonen et al., 2021). Therefore, there is a critical need for timely and accurate diagnostics, highlighting the need for innovative disease-modifying therapies, including pharmacological and non-pharmacological interventions for those at risk for dementia development.

The prodromal phase of dementia is most frequently characterized by *mild cognitive impairment* (MCI), corresponding to “mild neurocognitive disorder,” classified as 6D71 in ICD-11 (World Health Organization, 2019), and as 331.83 in DSM-5-TR (American Psychiatric Association, 2022). MCI refers to an objective impairment in cognitive functioning that goes beyond normal age-related changes yet does not significantly impact daily activities. Although individuals with MCI can remain stable, or revert to a cognitively intact state, the risk of conversion to dementia is notably high, and MCI is thus typically considered a transitional stage between normal cognitive changes associated with aging and dementia (Petersen et al., 2001). MCI is often classified into two clinical subtypes: amnestic MCI and non-amnestic MCI, each associated with different etiologies and outcomes, where the former is more often associated with a prodromal dementia phase. Recently, biomarkers such as cerebrospinal fluid (CSF) analysis and positron emission tomography (PET) imaging have been introduced to detect amyloidopathy and tauopathy *in vivo* (Jack et al., 2018). While biomarker positivity has been suggested to identify the MCI prodromal to AD and predict dementia onset, a significant proportion of cognitively unimpaired individuals with biomarker positivity do not progress over time (Dubois et al., 2021). Moreover, a substantial number of cognitively unimpaired individuals over the age of 70 years display β-amyloid pathology (Knopman et al., 2003). Therefore, biomarkers alone may not accurately identify preclinical AD if other measures of cognitive reserve and resilience are not considered. Importantly, MCI progression is influenced by several partially independent factors, including the presence of AD pathology, APOE ε4 status, and comorbid conditions (Katabathula et al., 2023). These factors collectively contribute to the diversity of MCI outcomes, emphasizing the need for new methods to understand the condition's etiology and progression.

Epidemiological studies generally agree that ~35–50% of individuals with MCI (without established neurodegenerative disease) will experience progressive cognitive decline and develop dementia within 3–5 years from symptom onset (Roberts et al., 2014; Vega and Newhouse, 2014). Indeed, the risk of dementia is over 20 times higher for individuals with MCI compared to the cognitively healthy elderly population. The annual conversion rate during the MCI phase of dementia progression ranges from 5 to 20%, depending on the diagnostic criteria used (Marcos et al., 2016). Consequently, MCI serves as the harbinger of most new dementia cases annually. However, current clinical investigations of MCI are both time-consuming and require significant expertise, and they possess limited power in predicting dementia risk. Moreover, the MCI population is heterogeneous concerning known risk factors and comorbidities, such as endocrine-metabolic diseases, cardiovascular disease, diabetes, tobacco and alcohol use, obesity, depression, dyslipidaemia, diet and sedentary lifestyle, and sensory impairments (Livingston et al., 2020). Addressing such modifiable risk factors is an essential preventive dementia care objective. Furthermore, once disease-modifying treatments will become available worldwide, an effective screening method for early diagnosis must be established. Currently, the clinical diagnosis of MCI is based on neuropsychological testing. While neuropsychological testing effectively predicts dementia progression in the later stages of MCI (Robert et al., 2006; Sarazin et al., 2007), it fails to differentiate individuals in the early phase.

Furthermore, MCI diagnostics suffer from poor inter-rater reliability and non-harmonized diagnostic guidelines (Dubois et al., 2014; Jack et al., 2018), and a lack of standardized biomarkers for large populations (Frisoni et al., 2013). Current clinical and instrumental diagnostics are neither cost-efficient, widely accessible, non-harmful, nor user-friendly. All state-of-the-art AD biomarkers (e.g., cerebrospinal fluid, CSF; magnetic resonance imaging, MRI; positron emission tomography, PET) present significant limitations. The widespread use of these methods remains restricted due to their low availability, perceived invasiveness, high costs, and various contraindications. Equitable technologies and procedures must be developed to address the rising demand due to the aging population (Bertens et al., 2019). Moreover, individuals with MCI who are completely independent in their daily activities and only have a partial risk of developing dementia should not undergo needless diagnostic procedures, unwarranted worries, and costly and risky treatments. Crucially, delayed MCI diagnosis and subsequent dementia confirmation hinder healthcare professionals from identifying the optimal intervention window for delaying or reversing the dementia onset.

Recent years have witnessed an accelerated development of promising blood biomarkers (Cicognola et al., 2021) and multimodal AI algorithms based on classical machine and novel deep learning (Hou et al., 2019; Qiu et al., 2022) to identify earlier opportunities for intervention in the disease progression. Nevertheless, these approaches are still used primarily in research settings and clinical trials, often without robust external validation in everyday clinical routine. Naturally, their successful deployment in clinical practice will require resource demanding feasibility and health technology assessments. Furthermore, a better understanding of how the blood biomarkers interact with individual clinical characteristics and tissue-level pathological changes at different disease stages is increasingly essential. Initial neurodegenerative changes occur years before structural changes can be detected using current imaging methods. In AD-type neuropathology, synaptic dysfunction stands as an early risk sign (Terry et al., 1991) causing brain network disturbances observable with electrophysiological methods (electroencephalography, EEG; magnetoencephalography, MEG). Indeed, recent EEG and MEG studies on MCI subjects have shown promising accuracy in predicting the conversion from MCI to AD (Pusil et al., 2019; Miraglia et al., 2020).

Here, we introduce AI-Mind^1^, a 5-year initiative funded by a European Research and Innovation Action (RIA, H2020-SC1-BHC-06-2020, no. 964220). AI-Mind's core objective and primary outcome is to develop and provide an automated instrument for standardized population-based screening of the progression risk from MCI to dementia using cost-effective and non-invasive measures. This risk assessment will allow healthcare systems to concentrate pharmacological, rehabilitative, and lifestyle initiatives on high-risk subjects. Leveraging AI to extract relevant features from functional brain network data derived from M/EEG, digital cognitive assessment, genetic testing (APOE), and blood biomarker information (P-tau), we aim to achieve the overarching goal of predicting the risk of dementia at an early and personalized stage. While the anticipated AI-Mind tools represent a novel technological advancement, their intent is to complement, rather than replace, existing diagnostic tools for dementia. The project also addresses the ethical, social, and practice-related implications of AI-based risk prediction, involving patients, their relatives, and health professionals, in order to ensure responsible implementation.

One of the primary endpoints of AI-Mind is to optimize the use of EEG in dementia risk assessment as an established, globally accessible, and cost-effective technology. The primary objective of the AI-Mind *Connector* is to generate representations of synaptic-neuronal dysfunction inferred from M/EEG using AI-supported approaches. This outcome will be enriched with results from computerized cognitive testing and other risk markers (genetics, blood biomarkers, and clinical characteristics), creating the AI-Mind *Predictor*. Based on the large sample of MCI subjects evaluated in AI-Mind, the *Predictor* will estimate the individual's risk of developing dementia. Two AI-based approaches in the design will be considered: first, classical machine learning (ML) models, known for interpretability, and deep learning (DL) models, which excel in performance at the potential cost of interpretability and explainability. The ultimate outcome aligns with improved patient care, resource optimization, and enhanced outcomes for individuals with an increased risk of dementia. This proactive identification of the potential trajectory toward dementia empowers patients by enabling referrals for early risk stratification and even consideration for novel pharmacological interventions, all aimed at offering a more hopeful future (Robinson et al., 2015). Earlier identification can also motivate proactive management of modifiable non-neurological risk factors, which, importantly, may lead to a significantly extended duration of the initial stages of cognitive decline, and thus slow the progression of dementia (Livingston et al., 2020).

The advancement of digital and virtual twin technologies has become increasingly crucial in addressing the pressing needs of personalized healthcare and medical research. In a planned effort to contribute to this advancement, the AI-Mind project aims to implement data simulation and synthetizing methodologies to generate so-called hybrid models, enabling virtual lab scenarios for investigating hypothesis-driven individualized intervention possibilities. This innovation not only paves the way for more timely clinical personalized investigations and treatment options but may also demonstrate the potential of such virtual labs in revolutionizing healthcare in a broader context. This collaborative effort is made possible by the establishment of a European clinical network, eBRAIN-Health, aimed at generating comprehensive patient datasets on a high-security cloud platform. Moreover, the possible fusion of DL algorithms from EBRAINS partners AI-Mind, the Virtual Epileptic Patient (Jirsa et al., 2017), and The Virtual Brain (Sanz Leon et al., 2013; Schirner et al., 2022), holds great promise for authentic human knowledge driven brain modeling. By personalizing simulations and dissecting large brain imaging datasets, these technologies may offer new insights into brain function and possibly innovative therapies for neurological disorders, guided by the combination of EEG data synthesis, real life high-quality data, and coordinated computational resources by the federated EBRAINS infrastructure. A detailed account of this coordinated action is not in the scope of the current article and will be documented in collaboration with the eBRAIN-Health project elsewhere as the initiative progresses.

In this paper, we will introduce the clinical study protocol of AI-Mind and present briefly some of the clinical, ethical, social, and technical challenges the project addresses. While mentioned superficially here, other aspects of the AI-Mind project, including the AI and electrophysiological methodology, and regulatory framework, will be discussed in depth in future publications.

## 2 Materials and methods

### 2.1 Clinical study design and sites

The study includes a clinical assessment (for inclusion), a baseline visit (V1), and three follow-up visits (V2, V3, and V4), each separated by 8 months. The clinical assessment is repeated at V4. Table 1 summarizes the procedures conducted at each visit. The five clinical centers involved in recruitment and data collection are the Department of Neurology, Oslo University Hospital, Norway (OUS); the Memory Clinic at the Catholic University of the Sacred Heart, Rome, Italy (UCSC-MC); IRCCS San Raffaele, Rome, Italy (IRCCS); Helsinki University Hospital, Finland (HUS); and Universidad Complutense de Madrid, Madrid, Spain (UCM). The AI-Mind research activities are conducted in parallel with national state-of-the-art (SOA) procedures for MCI and progressive dementia diagnostics. The study does not include any new pharmacological treatments.

**Table 1:** Research activities in the AI-Mind protocol.

**Procedure**	**Performed at time point (months since inclusion)**				
	**Inclusion (M0)**	**V1 (M0)**	**V2 (M8)**	**V3 (M16)**	**V4 (M24)**
128-channel resting-state EEG		X	X	X	X
Computerized cognitive testing (CANTAB^®^)		X	X	X	X
Blood sampling (APOE, p-tau)		X	OUS		X
MEG (at UCM and HUS only)		HUS			UCM, HUS
Sociodemographic data		X			
Global cognitive screening (MoCA/MMSE)	X		X	X	X
Clinical Dementia Rating (CDR)	X		X	X	X
Clinical interview (Case Report Form, CRF)	X				X
Registering data on pharmacological treatments	X		X	X	X
Self-administered memory questionnaire	X				X
Neuropsychological assessment	X				X

The clinical interview (summarized in a Case Report Form; CRF) comprises a record of the participant's anamnestic history, including risk factors, and somatic and psychiatric comorbidities, and scales for measuring depression, alcohol consumption, and activities of daily living (ADL).

APOE, Apolipoprotein E; CANTAB^®^, Cambridge Neuropsychological Test Automated Battery; CDR, Clinical Dementia Rating; EEG, electroencephalography; MEG, magnetoencephalography; MMSE, Mini Mental State Examination; MoCA, Montreal Cognitive Assessment; p-tau, plasma phosphorylated tau.

### 2.2 Participants and measures of progression

#### 2.2.1 Target sample and recruitment

The AI-Mind study aims to recruit 1,000 MCI participants aged between 60 and 80 years, consisting of 250 participants per each of the four involved countries. The target sample size was estimated with a statistical power analysis, assuming that the AI-Mind *Predictor* will achieve minimum specificity and sensitivity levels of 0.90–0.95 in the target population where the prevalence of MCI presenting a prodromal stage of dementia and that of other etiologies are both ~50% (Cowley et al., 2019).

As of 1 September 2023, close to 900 participants have been included since the recruitment initiation. The recruitment aims at equal distribution of participants across the four countries. Due to differences in the national healthcare systems in the participating countries, the outreach strategy varied among countries. In Finland and Italy, participants were recruited directly from the hospitals' outpatient clinics (HUS, UCSC, and IRCCS). In Spain (UCM), participants were recruited from various neurological clinics in the proximity of Madrid and from community centers. In Norway (OUS), participant outreach was conducted through online distribution of a self-assessment questionnaire of memory and other cognitive functions (The Finnish Medical Society Duodecim, 2021). Those unable to sign the consent form or fill in the questionnaire online were offered the alternative to complete the forms on paper. People who reported mild cognitive symptoms and increased worry over their cognitive status in the questionnaire were invited for a comprehensive clinical examination and neuropsychological assessment.

#### 2.2.2 Inclusion and exclusion criteria

The AI-Mind study inclusion and exclusion criteria are listed in Tables 2, 3. The inclusion criteria were based on the criteria suggested by Winblad et al. (2004) and Bondi et al. (2014). These criteria include cognitive concerns (subjective complaints), objective cognitive impairment (beyond expected norms), preservation of functional independence, and absence of dementia. For inclusion and exclusion, the candidates are clinically evaluated by health personnel at their respective clinical centers. The clinical evaluation comprise global cognitive screening (see below), and anamnesis for family history of dementia/neurodegenerative diseases, possible risk factors associated with cognitive decline, somatic comorbidities, psychiatric symptoms, including questionnaires for depression (Beck's Depression Inventory, BDI; Beck et al., 1961; or Montgomery–Åsberg Depression Rating Scale, MADRS; Montgomery and Asberg, 1979) and alcohol consumption (Alcohol Use Disorders Identification Test, AUDIT; Babor, 2001), and current pharmacological treatments. The objective cognitive impairment is determined through performance on standardized neuropsychological tests (see below): either >1.5 SD below the mean of the norm data in one test score (Winblad et al., 2004) or >1 SD below the mean in two test scores of the same cognitive domain or in three tests of all different domains (Bondi et al., 2014). Level of daily functioning is addressed with the (Instrumental) Activities of Daily Living (I/ADL; Lawton and Brody, 1969) scale or similar measures. The inclusion procedures (Table 1; M0) are carried out prior to V1 and they will be repeated at V4. Considering the importance of the neuropsychological evaluation for the study, only native speaker subjects were recruited to eliminate language bias in the cognitive testing.

**Table 2:** Criteria and assessment of MCI and dementia progression in the AI-Mind protocol.

**MCI criteria for inclusion**	**MCI assessment**	**Progression assessment**
- Cognitive concern reported. - Objective evidence of impaired cognitive domain (Petersen/Windblad and Jak/Bondi criteria). - Preserved independent functioning. - Not demented (cognitive changes do not significantly impede social function or work activities). - Presence of objective longitudinal decline for no more than 3 years. - Substantial vascular causes ruled out.	- CDR ≤ 0.5 - MMSE ≥ 25 or MoCA ≥ 17 - (I)ADL ≥ 70%	- CDR > 0.5 - MMSE < 25 or MoCA < 17 - (I)ADL < 70%

MCI, Mild cognitive impairment; CDR, Clinical dementia rating scale; MMSE, Mini Mental State Examination; MoCA, Montreal Cognitive Assessment; (I)ADL, (Instrumental) Activities of Daily Living.

**Table 3:** Exclusion criteria in the AI-Mind protocol.

**Exclusion criteria**
- Documented history of cerebrovascular disease (i.e., major stroke episodes). - Clinically verified positive AUDIT (alcohol dependency) score. - Severe medical disorders associated with cognitive impairment (organ insufficiency, chronic infections, and endocrine disorders). - Severe head trauma with structural brain lesions and/or previous brain surgery. - Severe mental disorders (schizophrenia, known major depression, or bipolar disorder). - Neuroimaging evidence of other potential causes of cognitive decline (e.g., subdural haematoma, brain malignancy, and metabolic encephalopathy). - History of non-brain malignancy during the last 5 years. - Recent (< 3 month) introduction of psychotropic drugs including acetylcholinesterase inhibitors (AChEI) and/or memantine. - Participation in trials with experimental drugs.

AUDIT, Alcohol Use Disorders Identification Test.

#### 2.2.3 Global cognitive screening

The global level of cognitive performance is assessed with the Mini Mental State Examination (MMSE; Folstein et al., 1975) and the Montreal Cognitive Assessment (MoCA; Nasreddine et al., 2005), depending on the local practices. In AI-Mind, the cut-off scores for inclusion are MMSE ≥ 24 and MoCA ≥ 17 (see Table 2; Trzepacz et al., 2015).

#### 2.2.4 Neuropsychological assessment

Neuropsychological assessment is conducted to evaluate the MCI status. Performance is assessed in the following cognitive domains: memory, language, attention/executive function, and visual-constructive ability. Verbal and visual memory are tested with both active recall and recognition of a word list in the Rey Auditory Verbal Learning Test (RAVLT) and of a copied figure in the Rey-Osterrieth Complex Figure Test (ROCFT). Language functions are assessed with the Boston Naming test (BNT), as well as with the semantic and phonological Verbal Fluency (VF) tasks. Attention/executive function is assessed with the Trail-making test (TMT) A and B. Visual-constructive ability is assessed with the figure copying task in ROCFT. The selected tests have been demonstrated to differentiate between healthy aging, MCI, and dementia (e.g., Estévez-González et al., 2003; Ashendorf et al., 2008; Baerresen et al., 2015; Ellendt et al., 2017; McDonnell et al., 2020; Venugopalan et al., 2021). The tests are administered in the local language at each site. In Finland, due to the lack of applicable norms for RAVLT and ROCFT, the equivalent tests from the Consortium to Establish a Registry for Alzheimer's Disease (CERAD) battery are used. CERAD is administered on a separate day from the other neuropsychological tests. The neuropsychological assessment is administered at baseline and will be administered again at V4.

#### 2.2.5 Progression assessment

Table 2 lists the dementia progression evaluation criteria used throughout the project. At each visit, in addition to screening the participant's global cognitive status using either MoCA or MMSE, a clinical evaluation guided by the Clinical Dementia Rating (CDR) is conducted. CDR has a 5-point scale ranging from normal cognition to severe dementia. The score is based on the qualitative assessment of cognitive and functional status across six domains relevant to dementia. At each visit, a semi-structured interview focused on changes in cognitive functioning is conducted. The participant's responses are evaluated in comparison to information provided by a reliable informant (e.g., a family member or a close friend) and other qualitative and quantitative information available to determine a CDR score. If a participant has a stable CDR score at 1 or above at V2 or V3, the comprehensive neuropsychological assessment is conducted immediately (instead of at V4) to assess the progression of cognitive symptoms/conversion to dementia.

### 2.3 Neurophysiological recordings

#### 2.3.1 Electroencephalography

EEG is collected similarly at all five clinical sites. The recording protocol includes resting-state data acquired in four successive 5-min runs of alternating eyes-open and eyes-closed conditions. In addition, a standardized artifact registration run (harmonized in content and sequence across sites) is recorded prior to the resting-state data acquisition. The registered artifacts include instructed eye blinks, horizontal and vertical eye movements, head movements, facial muscle activity, chewing, coughing, yawning, and environmental auditory noise, and will be used to aid the automatic detection of these artifacts in the participant's resting-state data.

EEG is recorded at a sampling rate of 2,000 Hz with an anti-aliasing filter with a cut-off frequency of 520 Hz, using 126 cephalic electrodes prewired in an elastic cap (ANT neuro WaveguardTM), in addition to two auxiliary electrodes placed below the left eye (electro-oculogram; EOG), and on the right clavicle bone (electrocardiogram; ECG). In the evaluation of functional connectivity and network-based metrics, high-density EEG is advantageous compared to lower-density (Hatlestad-Hall et al., 2023). The electrodes are labeled according to the 10-5 system derived from the standardized 10-20 and 10-10 international systems (Oostenveld and Praamstra, 2001), and their impedances are kept as low as possible, preferably below 25 kΩ. The ground electrode is placed on the left mastoid, while the CPz electrode serves as the reference during recording. The signals are amplified by an eego™ mylab EE-228 amplifier system and digitalized and stored using eego™ software, both provided by ANT neuro/eemagine Medical Imaging Solutions GmbH, Berlin, Germany.

During the eyes-open recording, participants are instructed to sit in a relaxed position and maintain their gaze on the center of a cross that is positioned in front of them. They are instructed to avoid movement of the eyes and face muscles but are permitted to blink. For the eyes-closed recording, participants are instructed to refrain from falling asleep. On the day of the EEG recording, the participants are asked to minimize their intake of products containing caffeine.

All the EEG data, independent of the site of acquisition, will be analyzed similarly. The data will be preprocessed using an automated pipeline developed during the project, which includes detection and management of signal noise and prominent artifacts. Various methods for data cleaning are to be evaluated *post-hoc* by assessing their impact on the AI model performance. The similarity of measurement devices and protocols will minimize the data variability across sites, facilitating the harmonization of the data. It is important to note that the level of environmental electromagnetic noise may still vary across measurement sites. This mimics the every-day clinical practice, and thus any algorithm developed in the project should generalize over such differences.

#### 2.3.2 Magnetoencephalography

Simultaneous EEG and MEG data is collected at two clinical sites (HUS and UCM). The MEG recordings are performed in magnetically shielded rooms with corresponding 306-channel whole-head MEG devices at each site (Elektra Neuromag TRIUX in HUS, and TRIUX neo in UCM, both manufactured by MEGIN Oy, Helsinki, Finland). The MEG devices consist of triplet sensors of two planar gradiometers and one magnetometer at 102 locations, with each gradio-/magnetometer coupled to a Superconducting Quantum Interference Device (SQUID). Five head-position-indicator (HPI) coils are attached to the EEG cap to enable continuous head position monitoring during the measurement, and one bipolar EOG and ECG electrode and separate ground are applied. Data is collected at a sampling rate of 2,000 Hz and using an online band-pass filtered between 0.01 and 660 Hz. The MEG recording protocol is the same as that used for the EEG alone. The synchronization of the simultaneous EEG and MEG measurements is conducted using trigger pulses generated by the MEG system at given time intervals. MEG recordings will be acquired at V1 and V4 at HUS, and at V4 at UCM. Due to the global scarcity of the technology, MEG data does not constitute a mandatory input to the AI-Mind algorithms. The inclusion of MEG at two of the study sites is primarily motivated by scientific interest, enabling comparative analysis between MEG and EEG signals.

#### 2.3.3 Co-registration with template anatomical MRI data

To mimic current clinical practice of many countries, individual MRIs are not collected within the project. Instead, MRI templates are used to facilitate data analysis at the source level. To enable co-registration with, and adaptation of, MRI templates, individual EEG electrode positions are obtained from each participant by recording a 3D scan using a Structure Sensor (Mark II) ST02B™ scanner (XRPro LLC (Structure), Boulder, CO, USA) attached to an Apple iPad™ (7th Gen, Apple Inc., Cupertino, CA, USA), and software Scanner—Structure SDK™ (XRPro LLC). The participant's nasion and preauricular points are marked for anatomical referencing. In MEG, digitization of the HPI coils, anatomical landmarks (nasion/nose, preauricular points/ears), and a subset of the EEG electrodes (seven electrodes included in the 10-20 system) is conducted with a FASTRAK digitiser (Polhemus Inc, VT, USA, provided by MEGIN Oy).

### 2.4 Computerized cognitive testing

A set of the Cambridge Neuropsychological Test Automated Battery (CANTAB^®^ Cambridge Cognition Ltd) tests is administered at each visit in conjunction with the M/EEG recordings. The CANTAB^®^ is a cloud-based system for cognitive testing that is validated for use on an Apple iPad. The CANTAB^®^ enables an operator-independent, technologically augmented, and relatively language- and culture-independent cognitive assessment. The AI-Mind study protocol employs six CANTAB^®^ tests including a motor screening task (Table 4). The tasks were selected based on the recommendations by the supplier to probe cognitive functions, including memory and executive processing, that are typically affected in neurodegenerative conditions such as AD. In particular, the Paired Associates Learning (PAL) test has been reported to be sensitive with respect to discrimination between MCI, prodromal AD, and AD (Egerházi et al., 2007; Junkkila et al., 2012). All tests feature standardized voice-over instructions in local languages (Norwegian, Spanish, Italian, and Finnish/Swedish), which ensures accurate and consistent test administration across sites. The tests are administered in the tabulated order (Table 4; note that the delayed PRM task is presented after the SWM task), a session lasting ~45–50 min depending on the participant's performance and the speed at which the participant makes transitions between tests.

**Table 4:** CANTAB^®^ tests administered in the AI-Mind protocol.

**Full name**	**Cognitive domain and description**
Motor Screening Task (MOT)	Test measuring sensorimotor speed and skills. Supervised administration to ensure that the participant can perform the tests with respect to motor and visual functions
Paired Associates Learning (PAL)	Test for visual episodic memory and learning
Pattern Recognition Memory (PRM)	Includes both immediate and delayed recognition conditions. Test of visual pattern recognition memory in a 2-choice forced discrimination paradigm
Rapid Visual Information Processing (RVP)	Test for sustained attention
Spatial Working Memory (SWM)	Test for working memory and strategy
Delayed Matching to Sample (DMS)	Assesses visual matching and short-term visual recognition memory for non-verbalizable patterns

The tests are carried out in a fixed order; MOT, PAL, PRM (immediate), RVP, SWM, PRM (delayed), and DMS. The minimum time to elapse between the immediate and delayed conditions of PRM is 20 min.

### 2.5 Genetic and protein analyses

Blood samples for DNA isolation and plasma protein assessment are collected from participants at V1 and V2 (Oslo only), with no need for fasting; additional sample collections at V4 are currently planned. Collection and stocking procedures have been standardized via a Standard Operating Procedure. Currently, the APOE ε4 allele is the strongest genetic risk factor for AD (Corder et al., 1994; Lambert et al., 2009). The presence of this allele is associated with 10 to 30 times increased risk for both early-onset and late-onset AD. APOE ε4 status may significantly add to the value of EEG in predicting the disease progression in MCI subjects (Vecchio et al., 2018). In the AI-Mind *Predictor* model, the genetic data will be classified as increased risk or not. In addition to genetic biomarkers, we will measure plasma tau phosphorylated at threonine 217 (p-tau217) and plasma tau phosphorylated at threonine 181 (p-tau181). These blood-based biomarkers are associated with AD tau pathology. Emerging data indicates that these could also be promising screening tools to identify individuals with underlying amyloid and AD tau pathology (Janelidze et al., 2020; Ashton et al., 2022).

### 2.6 Data governance

**Figure 1:**
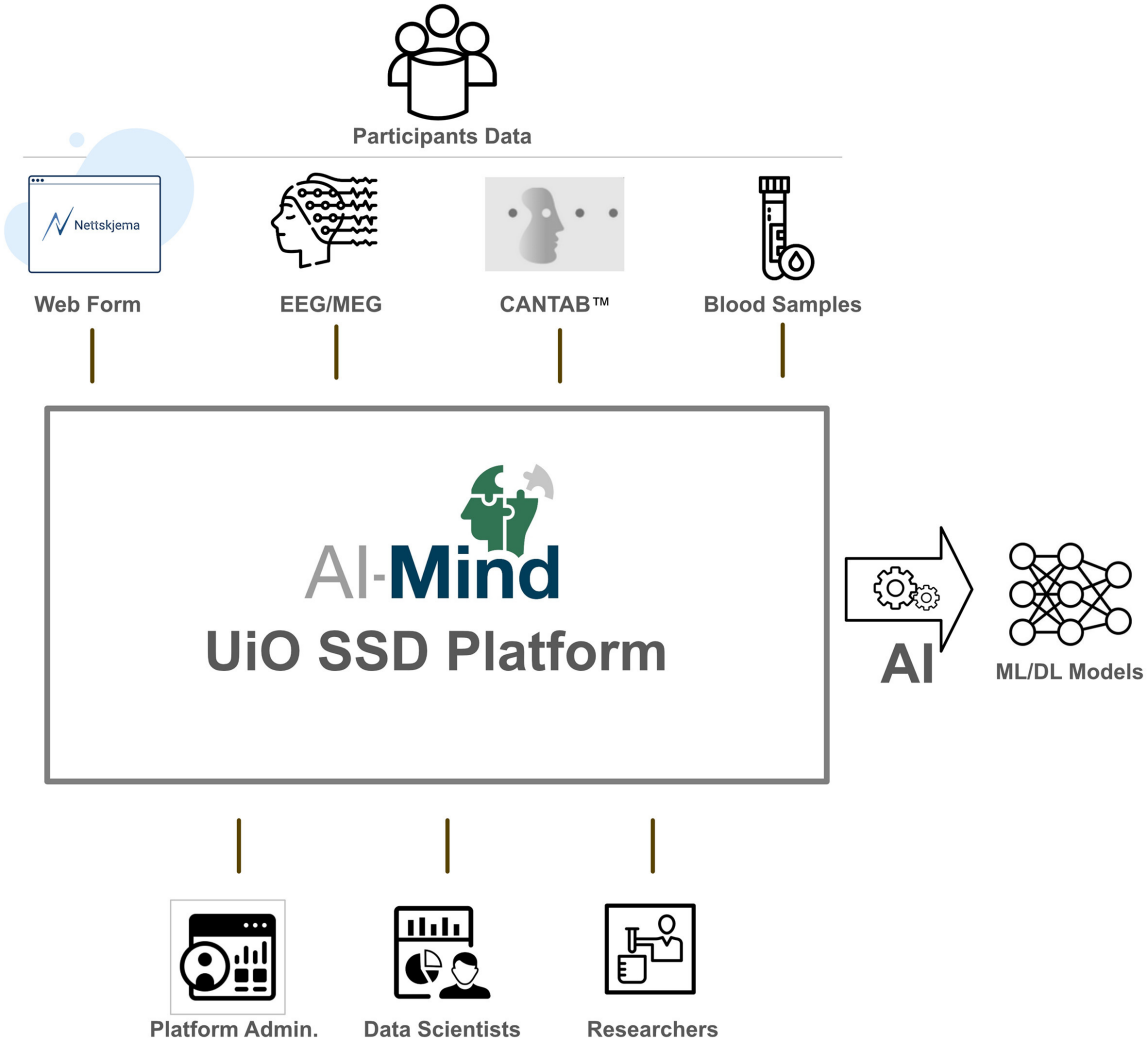
High-level AI-Mind study data flow.

All data (see Table 1) is stored centrally (Services for Sensitive Data; SSD) at the University of Oslo (UiO). Metadata, both clinical (CRF, CDR), sociodemographic, and technical (logs), are collected with the UiO service *Nettskjema* (“web form”)^2^. M/EEG data and the CANTAB^®^ data from Cambridge Cognition's storage service, are imported to the SSD storage area daily. The blood samples are shipped in bulk (~125 participants per shipment) to Oslo University Hospital, Norway, where all samples are stored in the AI-Mind project-specific biobank. Once analyzed, the APOE allele and p-tau data are stored in the project's SSD area. At the file staging area of the AI-Mind platform (located inside the SSD infrastructure), developed during the project, the data are verified, curated, re-processed (if applicable), and prepared for AI model development. A high-level view of the AI-Mind study data flow is shown in Figure 1.

### 2.7 Machine (deep) learning models: AI framework backend

The *Connector* and *Predictor* will be developed using data from AI-Mind's clinical study, as described above. The *Connector* utilizes M/EEG data, while the *Predictor* augments *Connector* output with additional modalities, including CANTAB^®^ scores, genetic and protein analysis results, to predict dementia progression risk. The dataset enables optimal models for both tools. For the machine learning, we aim at balancing the training and test data sets over gender, age, education level, APOE ε4 allele status, and use of acetylcholinesterase (AChE) inhibitors or memantine within each country. In the following, we briefly outline our development strategy for the AI-Mind tools.

The *Connector* identifies dysfunction based on spatiotemporal M/EEG measures. Its development will consider a wide range of established M/EEG features derived from multivariate time series and frequency data, and adjacency matrices obtained from functional connectivity analysis. These include power spectral density, synchronization measures (e.g., phase locking value and amplitude envelope correlation), and graph theory metrics (e.g., clustering and efficiency). Both classical ML and DL methods are evaluated. Classical ML, including Bayesian approaches (Gillberg et al., 2016; Leppäaho et al., 2019), incorporating prior expert knowledge is used as a benchmark for comparison to more expert-independent approaches based on the DL paradigm. Relevant DL approaches to M/EEG data include computer vision-based models and complex network analyses like Graph Neural Networks, Generative Adversarial Networks, and Convolutional Neural Networks. A detailed account of AI-based approaches to M/EEG analysis is beyond the scope of the current article (for a comprehensive review, see Roy et al., 2019). Future publications from the AI-Mind project will detail its AI-related development.

AI-Mind faces a shortage of labeled data crucial for ML and DL model training due to ongoing data collection. To address this, the models initially rely on unsupervised techniques such as clustering and dimension reduction. As more labeled data becomes available, the focus will shift to semi-supervised learning, improving the models' overall performance. Consequently, we anticipate that the AI framework of AI-Mind will increase its performance over time. The *Predictor* incorporates additional data to the *Connector* output, merging genetics, biomarkers, cognitive assessment, and demographic data, using DL-based dimensional reduction. This enables comparing “new” DL-identified variables with “conventional” variables.

Model bias arises from data imbalances in which underrepresented groups are excluded and needs to be addressed. Model variance occurs with scarce data in the subgroups, and outcome noise stems from unconsidered variables affecting predictions. To counter this, we will identify subgroups that require more discriminative variables and incorporate known metadata, while maintaining an awareness that metadata are not necessarily unbiased (Vyas et al., 2020), and thus reduce negative effects on fairness across different demographic groups. We will employ tools such as IBM's Fairness 360 and clustering algorithms to discover the bias due to overfitting, limited subgroup data, or outcome noise. Importantly, the AI-Mind study implements several measures to minimize bias. Recruitment, data collection, and storage/transfer across countries have been harmonized. Representative samples from European MCI populations ensure a balanced dataset. Cross-validation is used in the classifier building, and an independent test set validates the model performance. As data will be collected from five different locations, using identical hardware but under different conditions, we will use leave-one-center-out techniques to ensure robustness to site-specific patterns in the data. Lastly, the models will undergo uncertainty assessment, with epistemic (parameter uncertainty due to data scarcity) and aleatory (randomness-induced) uncertainty analyses. Epistemic uncertainty for a given decision (per sample uncertainty) is assessed using mainly an ensemble of deep learning models. The overall model uncertainty will be evaluated using bootstrap and cross-validation methods.

### 2.8 Model cards and digital interface: AI framework frontend

The AI-Mind's AI framework (AIF) includes a frontend interface. On the highest level, it takes AI-Mind data as input and generates model cards (Mitchell et al., 2019). These model cards inform the AI developer about model implementation and architecture, and later convey model details to users after the MCI prediction models are optimized. The AIF is designed to be automated and accessible to AI developers (and in the future, clinicians), comprising sequential steps.

1. Parameter and hyper-parameter definition, along with the choice of DL architecture. Users can modify these parameters and input additional information, such as ethical considerations and bias sources, which will be incorporated into the model card.
2. Preparation of “hidden” parameters that define the specific modeling architecture (for AI team's use).
3. Access to training and validation datasets, formatted uniformly for input.
4. Selection or parallel execution of different architectures and model types.
5. Training and testing of models, yielding performance metrics, loss curves, and (epistemic) uncertainty measures.
6. Compilation of model cards based on the preceding steps' outcomes.

For both *Connector* and *Predictor*, the AIF primarily provides a performance assessment of the models being tested, both for categorical and numerical outcomes. Additionally, in step 5 above, the *Connector* extracts mathematical expressions of learned features, which often represent linear combinations or functions of the original variables (M/EEG), offering insights into the original variable relevance and relationships.

### 2.9 Ethical approvals and informed consent

The national ethics committees in all countries with collaborating clinics approved the study and the related data transfer (documented in the AI-Mind Data Transfer Agreement). Participant data are pseudonymized in a standardized manner. Only the clinical center in which the participant is enrolled has access to individual identities. This access is regulated by national and institutional guidelines. Written informed consent is obtained from all study participants according to national and AI-Mind-specific guidelines posed by the European Commission. An Informed Consent tracking log has been created for each clinical site to record any further amendments in the potentially changing documents. Informed Consents are signed on paper in all sites, except for Norway (OUS) where the consent is signed digitally (participants who refuse digital signing for any reason are given the alternative to sign on paper). The signed documents are stored according to current local clinical site requirements, following the Personal Data Act/GDPR.

In Norway, the ethical committee considered the study results to have such important clinical implications that all Norwegian participants must be informed about individual test results. Consequently, each participant is not only registered in a research registry, but an individual electronic journal file has been created and will be monitored from the clinical perspective until the end of the project. Interestingly, between 50 and 90% of people report that they would be interested in knowing the results of a “predictive” or “reliable” test of their risk of developing dementia (Milne et al., 2018). Therefore, The AI-Mind project protocol includes the ethical and social implications of the use of this technology from the perspective of, and in relation to, current and future people with MCI and their relatives.

### 2.10 The AI-Mind consortium

AI-Mind is a multidisciplinary and public–industry consortium that brings together transdisciplinary hands-on expertise from clinics, industry, neuroscience research, and computer and data science in a multi-stakeholder setting. It consists of medical experts and opinion leaders on dementia, international experts in brain signal analysis and computer science, health technology assessment experts, small-medium enterprises (SMEs) and academic spin-off companies, one large industry organization, and patient and professional stakeholders. The consortium partners are listed in Table 5. Furthermore, AI-Mind maintains close collaborations with the European EBRAINS organization. Through a combination of our partners' expertise coupled with a strong stakeholder policy, we ensure that the AI-Mind tools will meet the regulatory and ethical standards for neurological AI diagnostic tools, while respecting European values and striving for quality and representativeness. AI-Mind promotes a unified EU-based diagnostic approach, addressing a major health challenge and supporting the development of an EU-based health system through path-to-market strategies.

**Table 5:** Alphabetical list of consortium partners.

**Partner name**	**Partner nationality**	**Partner main contribution**
Accelopment Schweiz AG	Switzerland	Project management
Aalto University	Finland	AI, machine learning
Alzheimer Europe	Luxembourg	Patient and user representation
BrainSymph AS	Norway	Medical technology, commercialization
Det Norske Veritas (DNV)	Norway	Data warehouse; data quality
Helsinki University Hospital	Finland	Clinical partner, neurophysiology
Scientific Institute for Research, Hospitalization and Healthcare, San Raffaele, Roma (IRCCS)	Italy	Clinical partner, neurophysiology
Lurtis Rules S.L.	Spain	Software architecture, commercialization
Neuroconnect Srl	Italy	Medical technology, commercialization
Oslo Metropolitan University	Norway	AI, deep learning
Oslo University Hospital	Norway	Project coordinator, clinical partner
Pre Diagnostics AS	Norway	Blood biomarkers, commercialization
Radboud University Medical Center	The Netherlands	HTA
Tallinn University	Estonia	User experience
Universidad Complutense de Madrid	Spain	Clinical partner, neurophysiology
Universitá Cattolica del Sacro Cuore	Italy	Clinical partner, HTA

AI, Artificial Intelligence; HTA, Health Technology Assessment.

## 3 Discussion

The AI-Mind project is a public health-orientated research and innovation initiative that aims at developing a screening tool for individuals concerned about their cognitive and brain health, and for determining the risk of dementia in mild cognitive impairment (MCI). As MCI serves as the precursor to a substantial majority of dementia cases, the accurate identification and timely treatment of MCI patients displaying an elevated risk of dementia progression have taken on an increasingly urgent significance. This imperative is particularly pronounced in the face of the global aging pandemic. Presently, both subjective concerns regarding cognitive function and objective cognitive impairments are often erroneously disregarded and attributed to normal aging processes rather than considered for further assessment (Sabbagh et al., 2020). This situation is partially rooted in the prevailing European clinical practice, which mainly relies on individual medical practitioners' judgments. The conventional approach to clinical investigations, involving extensive neuropsychological evaluations, has historically posed a bottleneck for healthcare systems, contributing to extensive waiting lists on a global scale.

To address these issues, the AI-Mind initiative conducts a comprehensive longitudinal clinical study focusing on subjects with MCI. Based on this study, the AI-Mind project develops two AI-based diagnostic support tools, namely, the *Connector* and the *Predictor*. The cornerstone of this endeavor is the construction of an extensive and standardized dataset, carefully assembled according to the outlined study protocol. This dataset is key in the realization of the project's objectives. Furthermore, the standardized and automated *Connector* and *Predictor* tools are designed to streamline the application of these methodologies in future investigations. This includes their potential application, e.g., in cross-cultural validation with diverse MCI populations. By deploying these tools, the AI-Mind initiative aims to contribute to a future of enhanced diagnostic capabilities and improved management strategies for individuals facing cognitive health concerns.

AI is increasingly considered a key technology to enable more efficient prediction of neurodegenerative disease risk in near-future healthcare. However, the technical, ethical, and societal challenges associated with the emergence of this new medical technology have not been sufficiently investigated, including their safe use in routine clinical activities and public health programmes (Rossini et al., 2022; Schmitz-Luhn et al., 2022). While the AI methods are capable of classifying meaningful patterns from large amounts of data, naïve integration of AI into traditional clinical work cannot camouflage the inherent limitations posed by such algorithms' input. Model input is susceptible to adverse influences, which in a medical setting may include unclassified comorbidities and inequal clinical resource access. Thus, despite recent advancements combining AI with classic diagnostic tools for predictive medical purposes in the industrialized world, its use comes with significant ethical challenges. For example, while the introduction of AI in radiology and nuclear medicine is considered a success, the accessibility of 1.5T MRI scanners varies substantially, e.g., 0.35 units per 100,000 in western countries compared to < 0.0004 units per 100,000 in Africa (Jalloul et al., 2023). The global distribution of PET scanners is even more concerning. On the other hand, lumbar puncture for CSF biomarker analysis is often perceived as invasive and poses a greater risk for adverse events than other procedures for dementia diagnostics. Thus, while promising in terms of accuracy, existing tools for dementia diagnostics, even when AI-supported, are not necessarily eligible for implementation as population-based screening methods (for a review, see Stephan et al., 2010). Furthermore, most of the current research of AI in healthcare focuses primarily on technical limitations and uncertainty, while the ethical and social dimensions of prediction accuracy are often ignored. It is crucial to consider the inevitable impact of algorithmic performance on global health responsibilities. Aside from pharmaceutical sponsored trials, few initiatives have been based on public-health oriented approaches and considered global transfer possibilities, with some exceptions (Rossini et al., 2019).

Although technical challenges have received considerably more attention than the social and ethical aspects of AI in healthcare, AI technology remains in its infancy with regards to medical applications. The training of classification models in classical machine learning typically relies on vast databases of labeled data. However, in medicine, most big data do not meet such requirements, in part due to the lack of standardized data infrastructure, but also because of medical data's inherent complexity. Another challenge pertaining to AI in medicine is the adaptation of AI architectures to the plethora of medical data modalities. In AI-Mind, the principal modality of investigation is time-series data derived from EEG. While classical machine learning techniques have demonstrated promising results on diagnostic classification based on features selected by human experts (e.g., Maestú et al., 2015; Vecchio et al., 2020), relatively little research has applied unsupervised deep learning techniques to raw EEG data (for a review, see Roy et al., 2019). In the AI-Mind study, as outlined previously, both classic machine learning and deep learning strategies will be employed, bearing in mind that both these families of techniques must tackle the challenge of transparency and explainability for their final use as supportive clinical decision-making tools.

Prognosis is intrinsically more complex than the final diagnosis in the context of AI, as it involves a projection into the future and is thus inherently uncertain. For AI to evolve and improve healthcare, the solutions developed must be perceived as trustworthy by patients, caregivers, health professionals, and other stakeholders. In AI-Mind, trustworthiness will be considered methodologically and ethically from three perspectives: explainability, fairness, and uncertainty quantification. *Explainability*, defined as a facet of transparency by the European Commission-appointed High-Level Expert Group on Artificial Intelligence, requires that a decision made by an AI system can be traced and understood by a human (HLEG on Artificial Intelligence, 2019). In addition to novel and transparent technical solutions, explainability involves an interdisciplinary effort between experts in medicine and AI. *Fairness* deals with the prevention of biased decisions to ensure an equal and just distribution of both benefits and costs, and that individuals and groups are free from unfair bias, discrimination, and stigmatization. Importantly, biased AI decisions can arise as a consequence of using non-representative data for model development or algorithmic bias. In AI-Mind, the former source of bias is mitigated by careful allocation of holdout data for internal model validation to the representativeness of our test data set, while algorithmically embedded bias will be addressed using various bias mitigation techniques. The AI development carried out in the AI-Mind project closely follows the principles outlined in the “White Paper on Artificial Intelligence: a European approach to excellence and trust” (European Commission, 2020). Importantly, to develop explainable and non-biased AI models, the features and their algorithmic classification, and the relative contributions that lead to decision making must be understood. For this purpose, we are investigating basic biological characteristics, for example, sex and age, to elucidate their impact on the predictive power of diagnostic AI models. Preliminary results indicate that in a deep learning context, EEG can be accurately classified according to sex and age. This and related findings (Jochmann et al., 2023) suggest that such factors may constitute biases in AI-supported decision making based on EEG data. The third technical element of trustworthiness considered in AI-Mind is *uncertainty quantification*, which is an aspect often disregarded when AI-supported decision-making systems are used. Importantly, uncertainty quantification in AI-supported systems has been shown to increase the accuracy of human decision-making (Abdar et al., 2021). Quantification of uncertainty will not only lead to more secure AI-supported human decision-making but can also aid prioritizing.

In addition to communicating the technical description of predictive AI tools in diagnostics, AI-Mind aims to direct attention to and facilitate the discussion of the socioeconomic and ethical aspects of such technology deployment, and its potential impact on the global society. It is our intention that AI-Mind should stimulate the future clinical use of AI-supported decision-making tools in European and other hospitals in a reliable and trustworthy manner. In collaboration with both global and EU-based scientific communities, included in the European infrastructure for sensitive data^3^ and the eBRAIN-Health infrastructure^4^, we aim to deliver quality AI model training data. The data will thus be accessible for future intervention studies in a virtual lab scenario, where the AI-Mind data and algorithms may be combined with simulation and synthesis technologies to effectively mitigate the technological limitations of deep learning and the scarcity of human expertise. By enhancing accuracy and efficiency, hybrid models may elevate medical diagnosis precision, enabling tailored treatment strategies based on individual needs. This paradigm shift augments outcomes for patients confronting neurological diseases' complexity. Furthermore, this initiative offers hospitals and clinical research groups the opportunity to test our tools, adapt and improve our algorithms, and transparently conduct performance comparisons in new cohorts using a federated learning approach. These opportunities are facilitated by the continuous integration of AI-Mind into the EBRAINS infrastructure, where maintaining compatibility with its Knowledge Graph and openMINDS metadata schema^5^ is a key target. Thus, convenient and secure data reuse is guaranteed, and future needs, such as cross-cultural algorithm adaptation, are facilitated.

Developing and implementing AI tools in healthcare raises legal concerns, including liability, patient rights, data and subject protection, health technology assessment (HTA) requirements, medical device regulations, and sustainability requirements. AI-Mind is committed to the content of the European AI strategy (European Commission, 2020), and adapts to the future liability and legislation framework of the European Union, which is a probable scenario during the project period. To bring the AI-Mind innovations into the clinics, it is crucial for the tools to meet these requirements, both in each nation and on the level of the European Union. Furthermore, the development and implementation of AI-Mind decision-support tools will require extensive processing of personal data inside a secure data framework. AI-Mind data processing strictly adheres to the General Data Protection Regulation (GDPR) of the European Union, in addition to any applicable national legislation concerning data processing and privacy issues. This compliance is guaranteed internally by the project's use of the University of Oslo's SSD server services, as well as within the EBRAINS infrastructure. All medical devices used in the AI-Mind study have been routinely employed clinically for several decades. All device deficiencies (including malfunction, user errors, and inadequate labeling) are documented and reported by the local investigator throughout the clinical investigation and appropriately addressed by the management support team of the AI-Mind project.

To meet the United Nations' sustainable development goals for the 2030 healthcare agenda (United Nations, 2015), the EU aims to create an ecosystem of excellence with a strong value chain involving research, innovation, and eventually stronger exploitation. The European Commission's White Paper on Artificial Intelligence (European Commission, 2020), the OECD's High-Level Expert guidelines published in April 2019 (OECD Legal Instruments, 2019) and the WHO's ethical considerations for health policy and systems research [Alliance for Health Policy and Systems Research (WHO) with the Global Health Ethics Unit (WHO), 2019] stress the need for value-based research and innovation during the expansion of AI-supported healthcare technologies. Proactive adaptation of traditional clinical MCI diagnostic procedures to the digital AI age is essential for the sustainability of European healthcare systems in the face of increasingly aging societies and limited health budgets. The AI-Mind *Connector* and *Predictor* will identify MCI patients who are at risk of dementia, and will thus facilitate timely preventive strategies in such individuals. On the other hand, the tools will also reduce the costs and burden of unnecessary investigations on low-risk individuals. Moreover, the timely selection of subjects to treat with novel innovative disease-modifying drugs, which are expensive and with a non-marginal risk of side-effects (i.e., brain oedema and hemorrhage), will benefit significantly from the development of the AI-Mind tools. Thus, the project will potentially have significant social and economic implications for both healthcare systems and those personally affected by MCI and dementia. Indeed, allowing for timely identification of at-risk individuals will pave the way for broader and faster access to adequate care and disease-modifying treatments, leading to improved patient care, optimized resource allocation, and better outcomes for individuals affected by MCI. Therefore, the expected academic output from AI-Mind will not only reflect scientific and technical insights in the context of AI-supported medicine, but also socioeconomic and ethical considerations for global health.

## 4 Conclusions

In this article, we have provided an overview of the AI-Mind initiative's background and its clinical study protocol. Going forward, the scientific publications from the AI-Mind project will expand upon the topics introduced here. These include describing M/EEG features, AI frameworks for AI-Mind data, managing data governance, addressing neuropsychological and cognitive aspects, examining health technology assessment (HTA), and ethical implications. Moreover, the clinical study outlined in this article is anticipated to significantly contribute to future discussions about the clinical assessment of AI-Mind across four European countries. This discussion will cover health, economic, and technological considerations. Presently, the project involves more than 70 associated employees on a regular basis, and it also includes representation from two major user groups: Alzheimer Europe and the International League Against Epilepsy. Importantly, neurological conditions are currently the leading cause of combined morbidity and mortality. This necessitates innovative, scalable, socioeconomically efficient, and sustainable solutions for our global society. With collective efforts, our consortium aims to introduce the AI-Mind platform and tools as a novel screening method for assessing dementia risk within the MCI population.

## Data availability statement

The original contributions presented in the study are included in the article/supplementary material, further inquiries can be directed to the corresponding author.

## Ethics statement

The studies involving humans were approved by Comitato Etico IRCCS San Raffaele Roma, Roma, Italy; Comitato Etico Fondazione Policlinico Universitario Agostino Gemelli IRCCS, Roma, Italy; Comité de Ética de la Investigación con Medicamentos, Hospital Clínico San Carlos, Madrid, Spain; HUS Regional Committee on Medical Research Ethics, Finland and Regional Comittees for Medical Research Ethics South East Norway. The studies were conducted in accordance with the local legislation and institutional requirements. The participants provided their written informed consent to participate in this study.

## Author contributions

IH: Writing – original draft, Writing – review & editing. CH-H: Writing – original draft, Writing – review & editing. CM: Writing – review & editing. HR: Writing – review & editing. FM: Writing – review & editing. JA-H: Writing – review & editing. SA: Writing – review & editing. VAn: Writing – review & editing. AA: Writing – review & editing. VAy: Writing – review & editing. ABa: Writing – review & editing. ABe: Writing – review & editing. CB: Writing – review & editing. RB: Writing – review & editing. NC: Writing – review & editing. CC: Writing – review & editing. EC: Writing – review & editing. AC: Writing – review & editing. SD: Writing – review & editing. RD: Writing – review & editing. AD-P: Writing – review & editing. AD: Writing – review & editing. GG: Writing – review & editing. JG: Writing – review & editing. PG-G: Writing – review & editing. DG: Writing – review & editing. TG: Writing – review & editing. HH: Writing – review & editing. MH: Writing – review & editing. LH: Writing – review & editing. JH: Writing – review & editing. SKa: Writing – review & editing. RK: Writing – review & editing. ASK: Writing – review & editing. AMK: Writing – review & editing. SKu: Writing – review & editing. DL: Writing – review & editing. ML: Writing – review & editing. PL: Writing – review & editing. AMD: Writing – review & editing. SMa: Writing – review & editing. SMe: Writing – review & editing. FM: Writing – review & editing. JM: Writing – review & editing. VM: Writing – review & editing. AØ: Writing – review & editing. JO: Writing – review & editing. TP: Writing – review & editing. JP: Writing – review & editing. LP: Writing – review & editing. DP: Writing – review & editing. AP: Writing – review & editing. MR: Writing – review & editing. FR-T: Writing – review & editing. AR-P: Writing – review & editing. TS: Writing – review & editing. MS-C: Writing – review & editing. RS: Writing – review & editing. SS: Writing – review & editing. AS: Writing – review & editing. MTo: Writing – review & editing. PT: Writing – review & editing. TT: Writing – review & editing. MTv: Writing – review & editing. RU: Writing – review & editing. RV: Writing – review & editing. FV: Writing – review & editing. AY: Writing – review & editing. PR: Writing – review & editing.

## Footnotes

^1^ www.ai-mind.eu

^2^ https://nettskjema.no/?lang=en

^3^ www.healthdatacloud.eu

^4^ www.ebrain-health.eu

^5^ www.ebrains.eu/data/find-data/find-data
